# A Study of the Clinical Profile and Respiratory Index of Severity in Children (RISC) Score in Infants Admitted With Acute Respiratory Infections at a Tertiary Care Hospital

**DOI:** 10.7759/cureus.43100

**Published:** 2023-08-07

**Authors:** Bangalore Srinivas Pranathi, Shilpa Krishnapura Lakshminarayana, Dhanalakshmi Kumble, Ravichandra Kothur Rangegowda, Mallesh Kariyappa, Gayathri Devi Chinnappa

**Affiliations:** 1 Pediatrics, Bangalore Medical College and Research Institute, Bengaluru, IND

**Keywords:** resource-limited setting, risc score, pneumonia, mortality, infant, acute respiratory infection

## Abstract

Background

Pneumonia is a major infectious cause of mortality in young children worldwide. The Respiratory Index of Severity in Children (RISC) score was designed with the intent to provide an objective mean to quantify the severity of lower respiratory tract infection in young children based on their risk of mortality. Knowledge about the clinical profile of acute respiratory infections and the scoring system predicting the risk of mortality helps in modifying treatment strategies. This study was undertaken at a resource-limited, tertiary-care public hospital in southern India with the objectives of describing the clinical profile of infants admitted with acute respiratory infections and determining the association of the RISC score with mortality.

Method

This was a retrospective observational study conducted over six months. Case records of infants admitted with acute respiratory infections were reviewed. The socio-demographic and clinical details of each case were recorded. The RISC score was calculated using clinical parameters which included the history of refusal of feeds, oxygen saturation lower than 90%, chest in-drawing, wheezing, and low weight-for-age. The maximum score was six. Descriptive data was represented using mean, standard deviation, and percentage or proportion. The association between any two categorical variables was analyzed using the chi-square test. The differences between any two continuous variables were analyzed using the independent sample t-test. A p-value of < 0.05 was considered statistically significant.

Results

A total of 75 infants were admitted with a diagnosis of acute respiratory infection during the study period. Of these, 68 were included in the study. The mean age of infants was 6.69 ± 3.96 months; 58.8% were male, 41 (60%) were exclusively breastfed, and 51 (75%) were up-to-date immunized. Twenty (29.4%) infants had a history of exposure to indoor smoke. The majority (67.6%) had pneumonia. Nine (13.2%) were mechanically ventilated. The mean duration of hospital stay was 8.16 ± 5.45 days. Sixty-three (92.64%) infants recovered and there were five deaths. The presence of less than 90% oxygen saturation (p-value=0.004), a diagnosis of severe pneumonia (p-value <0.001), and the need for mechanical ventilation (p-value <0.001) were significantly associated with mortality. A statistically significant (p-value=0.001) association was observed between the RISC score and mortality.

Conclusions

Addressable factors like the absence of exclusive breastfeeding, partial-immunization status, exposure to indoor smoke, and malnutrition were observed in infants with acute respiratory infections, which reinforces the importance of protective and preventive strategies for the control of pneumonia. The RISC score was observed to be beneficial in predicting mortality in an infant with acute respiratory infection. Triaging and early identification of infants at risk of mortality using this score could be very helpful in initiating timely treatment to reduce mortality, especially in resource-limited settings.

## Introduction

Pneumonia is one of the major infectious causes of mortality worldwide, in children under five years of age. According to the World Health Organisation (WHO), in 2019, pneumonia contributed to 14% of all under-five deaths. Mortality due to pneumonia is highest in southern Asia and sub-Saharan Africa [1]. Intending to reduce the mortality from acute respiratory infections (ARI) in developing countries, the WHO has published guidelines and simplified protocols for the diagnosis and management of ARI [2]. Pneumonia can be prevented in children with simple interventions like timely immunisation, adequate nutrition, and addressing environmental factors. Also, pneumonia can be easily treated with antibiotics [1].

Globally, there is a decreasing trend of lower respiratory infections in children under five years of age. However, in India, pneumonia continues to be the prime infectious cause of under-five deaths [3]. To enhance the timely utilisation of healthcare services by the caregivers of children with ARI and facilitate appropriate treatment, the WHO has revised the guidance for classifying and treating childhood pneumonia at first-level health facilities and outpatient departments [4]. It is critical to continue understanding ARIs and related risk factors for prevention, timely management, and better outcome.

The Respiratory Index of Severity in Children (RISC) score was designed in a South African study on children aged less than 24 months. The score was designed from clinical parameters and was intended to provide an objective mean to quantify the severity of a lower respiratory tract infection based on the risk of mortality. The study observed that among the HIV non-infected children, the median RISC score was one which corresponded to a 0% risk of mortality [5]. Further studies were recommended for validating the RISC score in additional populations and different settings [5]. Recently, a study observed that the RISC score correlated with the WHO interpretation of chest radiographs in children aged one month to 12 years [6].

Knowledge about the ARI and RISC score at health facilities could help in understanding the outcome and revising the management strategies to reduce mortality. Currently, there is a limited number of studies on the RISC score, especially in infants with ARI. This study was undertaken at a resource-limited, public, tertiary care hospital with the objectives of describing the clinical profile of infants admitted with ARIs and analysing the association of the RISC score with mortality among these infants.

## Materials and methods

The study was conducted in the Department of Paediatrics at Vanivilas Hospital, Bengaluru, India. The hospital is attached to the Bangalore Medical College and Research Institute and is one of the largest, tertiary-care public referral hospitals in the southern Indian state of Karnataka. The hospital receives referrals from all over the state. Annually, about 2750-3000 children aged between one month and 18 years are admitted, with about 600-700 admissions pertaining to respiratory illnesses. Being a public referral hospital, it is resource-limited due to a high admission rate and shortage of human resources.

This was a retrospective observational study conducted over a period of six months, from February 2022 to July 2022. All infants aged two months to 12 months admitted during the study period with a diagnosis of ARI as defined by the WHO [4], identified from the hospital records, were included. Infants less than two months or greater than 12 months of age and those with a history of developmental delay, gastro-oesophageal reflux disease, congenital heart disease, congenital malformations of the respiratory tract, mucociliary disorders, poisoning, pneumonia, and HIV infection/exposure were excluded.

The case records of the included infants were reviewed retrospectively and the following data were collected: age, sex, history of fever, refusal of feeds, fast breathing, exposure to indoor smoke, feeding details, immunisation and socioeconomic status, oxygen saturation in room air, presence of severe/moderate acute malnutrition, tachypnea, chest in-drawing and wheezing at admission, need for antibiotics, mechanical ventilation, duration of hospital stay, and outcome (death/discharged). The national immunisation schedule [7] was used to assess the immunisation status and was further classified as up-to-date, partial, and unimmunised. The socioeconomic status was assessed and classified according to the modified Kuppuswamy classification [8]. The WHO growth charts [9] were used to plot the weight-for-age and weight-for-length. The infants were categorised as having moderate and severe acute malnutrition according to the national guidelines [10]. Tachypnea was defined as a respiratory rate of more than 50 per minute [2]. Infants with ARI were further classified as having no pneumonia, pneumonia, and severe pneumonia according to the revised WHO classification of ARI [4]. The RISC score [5] was calculated from the presence of a history of refusal of feeds, oxygen saturation lower than 90% in room air, chest in-drawing, low weight-for-age, and wheezing at admission.

**Figure 1:**
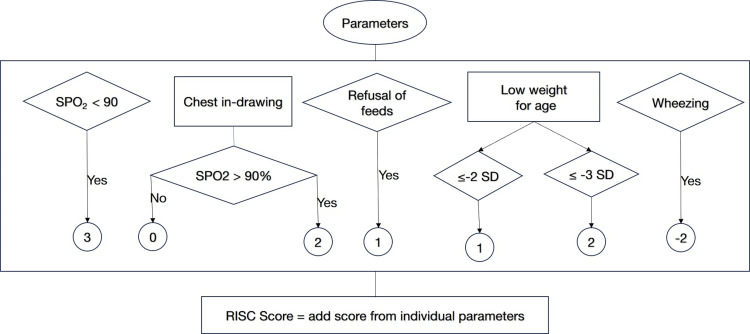
Respiratory Index of Severity in Children (RISC) scoring system SPO2: saturation of peripheral oxygen

Figure 1 describes the RISC scoring system. The presence of less than 90% oxygen saturation was given a score of 3. A score of 2 was awarded for the presence of chest in-drawing. A score of 1 was awarded for a history of refusal of feeds. Low weight-for-age (≤ −2 SD) and very low weight-for-age (≤ −3 SD) were given a score of 1 and 2, respectively. A score of −2 was given for the presence of wheezing. It is to be noted that a score of 2 for the presence of chest in-drawing was considered only if the oxygen saturation was greater than 90%. If the infant had chest in-drawing with less than 90% oxygen saturation, only a score of 3 was considered. The maximum score derived was 6.

The data were tabulated and statistically analysed using IBM SPSS Statistics for Windows, Version 20.0 (Released 2011; IBM Corp., Armonk, New York, United States). Descriptive data was represented using mean, standard deviation, and percentage or proportion. The association between any two categorical variables was analysed using the chi-square test. Differences among any two continuous variables were analysed using the independent sample t-test. A p-value of < 0.05 was considered significant.

## Results

A total of 75 infants were admitted with a diagnosis of ARI during the study period, of which 68 were included and seven were excluded (two cases with a history of developmental delay and one case each with gastro-oesophageal reflux, congenital heart disease, congenital malformations of the respiratory tract, accidental exposure to poison and pneumonia, and exposure to HIV). The clinical profile of infants admitted with ARI is shown in Table 1. The mean age was 6.69 ± 3.96 months and 40 (58.8%) were male. Fever was observed in 49 (72%) infants and was the most common presenting symptom. Only 41 (60%) were exclusively breastfed. Twenty (29.4%) had a history of exposure to indoor smoke and 51 (75%) were up-to-date immunised. The majority (n=61; 89.7%), belonged to socioeconomic classes three and four. Tachypnea and chest in-drawing were observed in 64 (94%) infants and were the most common signs. Oxygen saturation was less than 90% under room air in 27 (39.7%) infants. Thirty-three (48.5%) had wheezing. The weight-for-age was ≤ −2 SD and ≤ −3 SD in 20 (29.4%) and 15 (22%) infants, respectively. Fourteen (20.6%) and nine (13.2%) had severe and moderate acute malnutrition, respectively. Pneumonia was the most common classification and was observed in 46 (67.6%) infants; 64 (94.1%) received antibiotics and nine (13.2%) were mechanically ventilated. The mean duration of hospital stay was 8.16 ± 5.45 days. Sixty-three (92.64%) infants had improved and there were five (7.4%) deaths.

**Table 1:** The clinical profile of infants admitted with acute respiratory infections (n= 68) ARI: acute respiratory infections; SPO2: saturation of peripheral oxygen

Characteristics		Number	Percentage
Age	2-<6 months	36	52.9
	6-12 months	32	47.1
Sex	Male	40	58.8
History	Fever	49	72
	Fast breathing	27	39.7
	Refusal of feeds	18	26.4
	Exclusively breastfed	41	60.3
	Exposure to indoor smoke	20	29.4
Immuniation status [7]	Up-to-date	51	75
Socio-economic status [8]	Classes three and four	61	89.7
Signs	Tachypnea [4]	64	94.1
	Chest in-drawing	64	94.1
	Wheeze	33	48.5
	SpO_2 _<90% in room air	27	39.7
Weight-for-age [9]	≤ -2 SD	20	29.4
	≤ -3 SD	15	22
Weight-for-length [10]	≤ -3 SD, Severe acute malnutrition	14	20.6
	≤ -2 SD, Moderate acute malnutrition	09	13.2
Classification of ARI [4]	No pneumonia	04	5.9
	Pneumonia	46	67.6
	Severe pneumonia	18	26.5
Antibiotics	Received	64	94.1
Mechanical ventilation	Received	9	13.2
Outcome	Improved	63	92.64
	Death	5	7.4
Duration of hospital stay	<5 days	23	33.8
	6-10 days	27	39.7
	>10 days	18	26.4

The clinical profile of infants who died is shown in Table 2. Among the five infants who had died, four were aged between two to three months and all five had severe pneumonia with oxygen saturation less than 90% and needed mechanical ventilation. The duration of hospital stay was observed to be more than 10 days in three infants. Four infants had a RISC score of 6. 

**Table 2:** Mortality profile of infants with acute respiratory infection SPO2: saturation of peripheral oxygen; ARI: acute respiratory infection; RISC: Respiratory Index of Severity in Children

Clinical profile	Infant 1	Infant 2	Infant 3	Infant 4	Infant 5
Age in months	2	11	2	3	2
Sex	Female	Male	Male	Female	Male
Fever	Present	Present	Absent	Present	Absent
Fast breathing	Absent	Present	Present	Present	Present
Refusal of feeds	Present	Present	Present	Present	Present
Exclusively breastfed	Yes	No	No	Yes	Yes
Bottle feeding	No	Yes	yes	No	No
Exposure to indoor smoking	Yes	No	No	No	No
Partial immunization [7]	No	No	Yes	No	No
Socio-economic status [8]	Class three	Class four	Class three	Class four	Class three
Tachypnea present [4]	Yes	Yes	Yes	Yes	Yes
Chest in-drawing present	Yes	Yes	Yes	Yes	Yes
Wheeze present	No	No	No	No	No
SpO_2 _<90% under room air	Yes	Yes	Yes	Yes	Yes
Weight-for-age	< -3 SD	< -3 SD	< -2 SD	< -3 SD	< -3 SD
Weight-for-length [10]	Median	< -1 SD	Median	< -3 SD	< -3 SD
ARI classification [4]	Severe pneumonia	Severe pneumonia	Severe pneumonia	Severe pneumonia	Severe pneumonia
Received antibiotics	Yes	Yes	Yes	Yes	Yes
Mechanical ventilation	Yes	Yes	Yes	Yes	Yes
Duration of hospital stay in days	1	3	11	29	30
RISC score	6	6	5	6	6

The analysis of various factors for mortality is shown in Table 3. It was observed that the presence of less than 90% oxygen saturation at admission (p-value=0.004), diagnosis with severe pneumonia (p-value <0.001), and the need for mechanical ventilation (p-value <0.001) were significantly associated with mortality.

**Table 3:** Analysis of various factors with mortality in infants with acute respiratory infections * p-value for mortality calculated using the chi-square test SPO2: saturation of peripheral oxygen

Factors	Total	Improved	Death	p-value^*^
2-<6 months	36	32	4	0.206
6-<12 months	32	31	1	0.208
Bottle feeding	24	23	1	0.4
Partial immunisation status	17	16	1	0.789
Class four socio-economic status	30	28	2	0.8
Moderate and severe acute malnutrition	14	21	2	0.420
Mechanical ventilation	9	4	5	<0.001
Severe pneumonia	18	13	5	<0.001
Hospital stay of more than 10 days	18	15	3	0.016
SpO_2_ <90%	27	22	5	0.004

The RISC score in infants admitted with ARI is shown in Table 4. Twenty-two infants had a RISC score greater than or equal to 5.

**Table 4:** The Respiratory Index of Severity in Children (RISC) score in infants admitted with acute respiratory infection

RISC Score	Number of Infants (%)	Improved	Death
≤1	9 (13.2)	9	0
2	11 (16.1)	11	0
3	14 (20.5)	14	0
4	12 (17.6)	12	0
≥ 5	22 (32.3)	18	5

The association of the RISC score with mortality is shown in Table 5. The mean RISC score among infants with mortality was 5± 0.4472. There was a statistically significant correlation between the RISC score and mortality (p-value= 0.001). 

**Table 5:** Independent sample t-test showing association of the RISC score with mortality ^^^ T statistics; ^#^ Degree of freedom * p-value for mortality calculated using the independent sample t-test RISC: Respiratory Index of Severity in Children

	Mortality								
	Yes			No					
	N	Mean	SD	N	Mean	SD	T^^^	Df^#^	p-value*
RISC score	5	5.8	0.4472	63	3.2857	1.8874	2.952	66	0.0043

## Discussion

Prematurity, history of measles, incomplete immunisation, congenital heart disease, living in a kutcha house, poor ventilation, overcrowding, and open defecation have been observed as risk factors for severe pneumonia [11]. Also, young age and malnutrition have been recognised as independent risk factors for pneumonia [12]. In this study, the mean age of infants was around six months and about one-third had a history of exposure to indoor smoke and partial immunisation status. Exclusive breastfeeding was observed in only three-fourths of the cases. Bottle-feeding practice was observed in a significant number of cases and also about 50% of cases belonged to socio-economic status classes 4 and 5. One-third of cases had acute malnutrition. Young age, malnutrition, and unimmunised or partially immunised status have been observed as independent risk factors for mortality [13]. Also, mechanical ventilation and prolonged hospital stay have been significantly associated with mortality in children with severe pneumonia [14]. In this study, the majority of cases who died were between two to three months of age, and the presence of less than 90% oxygen saturation at admission, severe pneumonia, and the need for mechanical ventilation were significantly associated with mortality.

The RISC score was formulated to assess the severity of ARI in children. It was derived from simple clinical parameters and is independent of radiological observations or laboratory measurements. Also, with a negative score for the presence of wheezing, the RISC score was intended to overcome the drawback in the WHO classification of pneumonia where there is a possibility of over-diagnosing pneumonia in children with wheezing [5]. A study that validated the RISC score for predicting mortality among Malawian children aged 0-24 months with pneumonia observed that at scores of three and four, the sensitivity and specificity in predicting mortality were 59%, 78%, and 32.6%, 93.1%, respectively [15]. Another study observed that at the cut-off level of three, the sensitivity and specificity of the RISC score in predicting mortality were 94.1% and 73.6%, respectively [13]. In this study, there was a statistically significant (p-value<0.001) association between the RISC score and mortality in infants admitted with ARI.

Although pneumonia is a preventable and treatable condition, it continues to be a substantial burden on the health of children. The integrated Global Action Plan for Pneumonia and diarrhoea emphasises a combination of protective strategies like promoting exclusive breastfeeding and adequate complementary feeding, preventive strategies like timely vaccinations, personal hygiene, and tackling pollution, and treatment strategies like timely and appropriate treatment [1]. The government of India is committed towards the control of pneumonia and emphasises on protective, preventive, and intervention strategies to control pneumonia. In 2019, the government of India released revised guidelines for the management of childhood pneumonia, which is based on the revised WHO classification of pneumonia [16].

This study shows that despite various efforts by the government towards pneumonia control, bottle feeding, partial immunisation status, exposure to indoor smoke, lower socio-economic classes, and acute malnutrition are still observed in children with pneumonia. These findings highlight the importance of creating awareness and implementing exclusive breastfeeding and appropriate complementary feeding, timely immunisation, and health education strategies across all levels of healthcare facilities. As timely diagnosis and treatment are critical, the RISC score could be useful in triaging and predicting the risk of mortality, thereby helping in planning interventions to reduce mortality, especially in resource-limited settings. However, the RISC score is not intended to reassess pneumonia cases or infants with hospital-acquired ventilator-associated pneumonia cases where radiological and laboratory investigations might aid in further management.'

The limitations of this study are that it was a retrospective study design and a single-centre experience. More prospective studies on the RISC score could validate this scoring system in our region so that it could be considered by policymakers for incorporation into the national guidelines for triaging patients with ARI.

## Conclusions

Addressable factors like the absence of exclusive breastfeeding, partial-immunisation status, exposure to indoor smoke, and malnutrition were observed in infants with ARI, which reinforces the importance of protective and preventive strategies for the control of pneumonia. The RISC score was observed to be beneficial in predicting mortality in an infant with an ARI. Triaging and early identification of infants at risk of mortality using this score could be very helpful in initiating timely treatment to reduce mortality, especially in similar resource-limited settings.
